# Sumatriptan ameliorates renal injury induced by cisplatin in mice

**DOI:** 10.22038/ijbms.2019.33620.8020

**Published:** 2019-05

**Authors:** Gholamreza Bazmandegan, Morteza Amirteimoury, Ayat Kaeidi, Ali Shamsizadeh, Morteza Khademalhosseini, Mohammad Hadi Nematollahi, Mahsa Hassanipour, Iman Fatemi

**Affiliations:** 1 Physiology-Pharmacology Research Center, Research Institute of Basic Medical Sciences, Rafsanjan University of Medical Sciences, Rafsanjan, Iran; 2 Non-Communicable Diseases Research Center, Rafsanjan University of Medical Sciences, Rafsanjan, Iran; 3 Clinical Research Development Center, Ali Ibn Abitaleb Hospital, Rafsanjan University of Medical Sciences, Rafsanjan, Iran; 4 Department of Physiology and Pharmacology, Rafsanjan University of Medical Sciences, Rafsanjan, Iran; 5 Department of Pathology, Rafsanjan University of Medical Sciences, Rafsanjan, Iran; 6 Department of Clinical Biochemistry, Kerman University of Medical Sciences, Kerman, Iran

**Keywords:** Cisplatin, Mice, Nephrotoxicity, Oxidative stress, Sumatriptan

## Abstract

**Objective(s):**

Cisplatin (Cis) is an anticancer compound, which is used for the treatment of various cancers. Sumatriptan (Suma) is a selective agonist of 5-hydroxytryptamine 1B/1D (5HT1B/1D) receptor, which is prescribed for the management of migraine. It is well-established that Suma has anti-inﬂammatory and antioxidant properties. We have explored the protective effects of Suma in the mitigation of Cis-induced nephrotoxicity.

**Materials and Methods:**

The mice received a single IP injection of Cis (20 mg/kg) on the first day of the experiment. Suma treatment (0.1 and 0.3 mg/kg/day, IP) was started on day 1 and continued for 3 consecutive days.

**Results:**

Creatinine (Cr), blood urea nitrogen (BUN) and malondialdehyde (MDA) levels were elevated and glutathione peroxidase (GPx) as well as superoxide dismutase (SOD) activities were decreased in Cis-treated mice. Suma (more potently 0.3 mg/kg) reduced Cr, BUN and MDA levels and increased SOD and GPx levels. Suma also reduced the acute renal injury (tubular degeneration, tubular cells vacuolation, tubular necrosis and cast), which corresponded to kidney damage in Cis-treated mice.

**Conclusion:**

These findings demonstrate that Suma mitigates Cis-induced renal injury by inhibition of oxidative stress and enhancing the antioxidant enzymes activities.

## Introduction

Cisplatin (Cis) is an antineoplastic drug, which is used in the treatment of different cancers (1). Renal toxicity is the most important side effects of Cis-based chemotherapy, which limits the usage of this drug in clinic (2). The mechanism of nephrotoxicity induced by Cis is not completely understood. However, oxidative stress and lipid peroxidation have been implicated as contributing factors (3, 4). It is well-established that overgeneration of reactive oxygen species (ROS) increases malondialdehyde (MDA) and decreases the enzymes of antioxidant system such as superoxide dismutase (SOD) and glutathione peroxidase (GPx) in renal tissue, which are the major alterations in Cis-induced nephrotoxicity (5, 6). It has been reported that Cis increases the blood urea nitrogen (BUN) and creatinine (Cr) (7, 8). Therefore, there is a need to pursue a compound that can efficiently diminish Cis-induced nephrotoxicity to improve the chemotherapeutic efficacy of Cis.

Sumatriptan (Suma), a selective agonist of 5-hydroxytryptamine 1B/1D (5HT1B/1D) receptor, is a well-tolerated drug for the management of cluster headache and migraine. Suma has minor and transient adverse effects (9, 10). Recent studies have introduced some biological activities, including anti-inﬂammatory and antinociceptive effects for Suma (11, 12). Suma induces direct antioxidant properties through scavenging the nitric oxide (NO) and ROS (13). It is well-established that Suma causes marked vasoconstrictor responses in kidney, which is followed by a secondary vasodilatation through release of NO (14). Moreover, the anti-inflammatory properties of Suma are mediated by reduction in the release of neuropeptides such as calcitonin gene-related peptide and substance P, which are effective against the inflammatory factors (15). 

Thus, the purpose of our study was to explore whether Suma affects the Cis-induced kidney damage in mice by studying oxidative stress indices and histological alterations. 

## Materials and Methods

***Animals***

Twenty-eight male mice (weighing 25-30 g, 6-7 weeks old) were used in this study. Animals were maintained under standard conditions (22±2 ˚C temperature; 12-12 hr light/dark cycle) with free access to standard laboratory food and purified drinking water. All procedures were conducted according to the ethical guideline of Rafsanjan University Research (Ethic code: IR.RUMS.REC.1396.85).

***Chemicals***

Suma was obtained from Sigma-Aldrich (Germany) and Cis (CISPLATIN MYLAN®) was obtained from MYLAN Company (France). Drugs were administrated intraperitoneally (IP), and Suma was freshly dissolved in normal saline (9% sodium chloride). 

***Experimental design***

Mice were randomly separated into four groups (n = 7). Group I (control group) received no treatment. Group II (Cis group) received Cis (20 mg/kg) on the first day of the experiment. Group III and IV (Suma 0.1 and Suma 0.3) received Cis on the first day of the experiment, and Suma at the doses of 0.1 and 0.3 mg/kg/day was started on day 1 and continued for 3 consecutive days. The route of administration and dosages were selected from previous works (16, 17).

***Sample collection***

On day 4 of the experiment, blood samples were collected from the orbital sinus under diethyl-ether anesthesia. The blood samples were centrifuged at 3000 rpm for 10 min to separate serum. The serum samples were stored at -20 ºC for measurement of BUN and Cr. Then, animals were sacriﬁced by rapid decapitation and both kidneys were immediately removed and washed with cold normal saline. For pathological studies, the left kidney was fixed in 10% neutral buffered formalin. For biochemical assessments, the right kidney was shock-frozen in liquid nitrogen and kept at –80 ^°^C.

**Figure 1:**
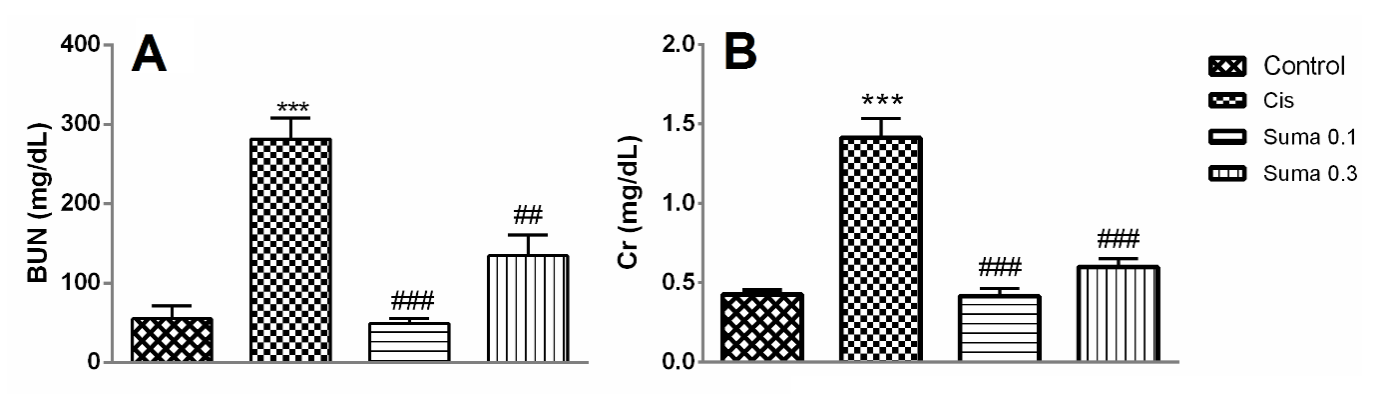
The effect of treatment with Suma on BUN (A) and Cr (B) levels in Cis-induced nephrotoxicity. Values are means±SEM (n=7). Mice received no treatment, Cis (20 mg/kg) or Cis and Suma (0.1 and 0.3 mg/kg for 3 consecutive days), and kidneys were harvested after 96 hr. * Significant difference in comparison with the control group (***P<0.001); # Significant difference in comparison with the Cis group (##*P*<0.01 and ###*P*<0.001). Cis: Cisplatin; Suma: Sumatriptan; BUN: Blood urine nitrogen; Cr: Creatinine

**Figure 2:**
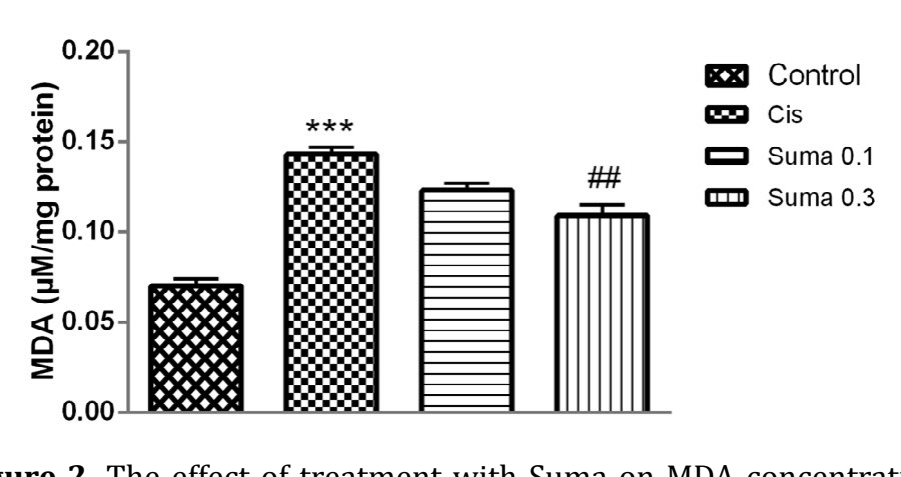
The effect of treatment with Suma on MDA concentration in Cis-induced nephrotoxicity. Values are means±SEM (n=7). Mice received no treatment, Cis (20 mg/kg) or Cis and Suma (0.1 and 0.3 mg/kg for 3 consecutive days), and kidneys were harvested after 96 hr. * Significant difference in comparison with the control group (***P<0.001); # Significant difference in comparison with the Cis group (##*P*<0.01). Cis: Cisplatin; Suma: Sumatriptan; MDA: Malondialdehyde

**Figure 3:**
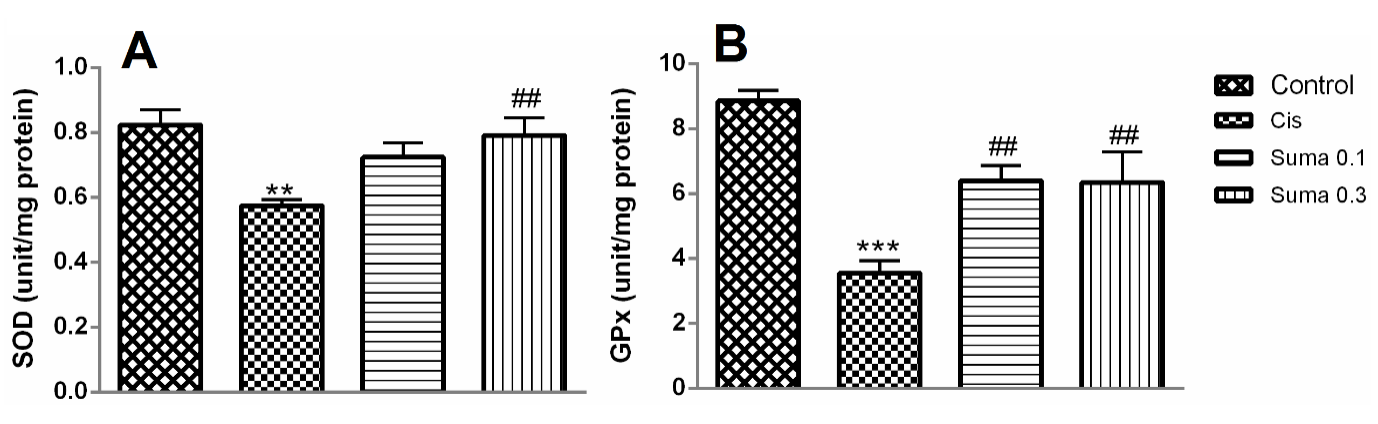
The effect of treatment with Suma on SOD (A) and GPx (B) in Cis-induced nephrotoxicity. Values are means±SEM (n=7). Mice received no treatment, Cis (20 mg/kg) or Cis and Suma (0.1 and 0.3 mg/kg for 3 consecutive days), and kidneys were harvested after 96 hr. * Significant difference in comparison with the control group (**P<0.01 and ***P<0.001); # Significant difference in comparison with the Cis group (##*P*<0.01). Cis: Cisplatin; Suma: Sumatriptan; SOD: Superoxide dismutase; GPx: Glutathione peroxidase

**Figure 4:**
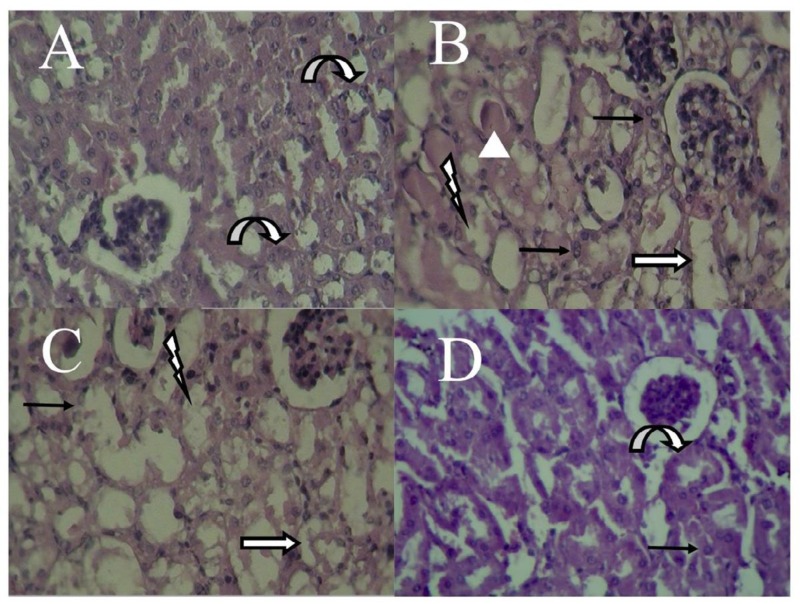
Histopathological observations showing the effects of Suma on Cis-induced nephrotoxicity changes in kidney. Mice received no treatment, Cis (20 mg/kg) or Cis and Suma (0.1 and 0.3 mg/kg for 3 consecutive days), and kidneys were harvested after 96 hr (kidney sections stained with Hematoxylin & Eosin, magnification X 400). (A) Control group; (B) Cis group; (C) Suma 0.1 group; (D) Suma 0.3 group. Cis: Cisplatin; Suma: Sumatriptan. Bent arrow: lumen of the normal collecting tubules; Lightning bolt: tubular degeneration; Line arrow: tubular cells vacuolation; White arrow; tubular necrosis; Triangle: cast

**Table 1:** Effect of sumatriptan on kidney histopathology in cisplatin-induced nephrotoxicity in mice

Groups	Histological criteria			
	Cast	Tubular necrosis	Tubular degeneration	Tubular cells vacuolation
Control	0±0	0±0	0±0	0±0
Cis	1.83±0.30**	2.16±0.3***	2.83±0.16***	2.16±0.16*
Suma 0.1	0.33±0.21#	1.16±0.16	1.33±0.21	1.16±0.30
Suma 0.3	0.16±0.16##	0.5±0.22#	0.66±0.21#	0.42±0.20#

Values are means ± SEM (n = 7).

Cis: Cisplatin; Suma: Sumatriptan.

* Significant difference in comparison with the control group (*P<0.05, **P<0.01 and ***P<0.001)

# Significant difference in comparison with the Cis group (#*P*<0.05 and ##*P*<0.01).

***Serum parameters***

The serum levels of BUN and Cr were measured using the biochemical autoanalyzer (MINDRAY, China) with respective commercial kits (Bayer S.A. Diagnostic).

***Biochemical assays***

The kidneys were defrosted, homogenized with phosphate-buffered saline (1/10 w/v) and centrifuged at 6000 rpm for 20 min in 4 ^°^C. The supernatants were collected for the biochemical assays such as MDA level and SOD and GPx activities using commercial kits specific for mice (ZellBio, Germany).

***Pathological evaluations***

For pathological assessment, the kidney samples were fixed in formalin (10%), then kidney samples were dehydrated, embedded in paraffin and sectioned (5 µm). The sections were stained with hematoxylin & eosin (H&E) and observed under a light microscope (Nikon Labophot, Japan) by an expert pathologist for the presence of tubular cells vacuolation, tubular degeneration, tubular necrosis and cast (18). The severity of these indices was scored into 4 categories: normal=0, mild=1, moderate=2 and severe=3 and the average values were reported (19).

***Statistical analysis***

Graphpad Prism software version 6 was used for statistical analysis (Graphpad Software, USA). The results are expressed as mean±SEM. Differences between groups were determined using ANOVA followed by Tukey’s *post hoc* analysis. Non-parametric variable (pathological indices) were analyzed by Kruskal-Wallis test followed by Dunn’s *post hoc* analysis. *P* value less than 0.05 was considered to be significant.

## Results

***Renal function***

As shown in Figure 1, the levels of Cr and BUN significantly increased in the Cis group in comparison with the control group (*P*<0.001 in all groups). Administration of Suma at both doses (0.1 and 0.3 mg/kg) significantly decreased the BUN levels in Cis-administered mice (*P*<0.001 and *P*<0.01, respectively) (Figure 1A). Moreover, treatment with Suma (0.1 and 0.3 mg/kg) for 3 consecutive days significantly decreased the Cr in Cis-administered mice (*P*<0.001 in all groups) (Figure 1B). 

***Renal lipid peroxidation***

Our results indicated that administration of Cis significantly increased the concentration of MDA in the Cis group compared to normal mice (*P*<0.001) (Figure 2). On the other hand, treatment with Suma (0.3 mg/kg) significantly reduced MDA level in comparison with the Cis-administered mice (*P*<0.01). 

***Renal antioxidant enzyme activities***

The SOD and GPx activities were significantly reduced in the Cis group compared to the normal mice (*P*<0.001 in all groups) (Figure 3). Moreover, Suma administration (0.3 mg/kg) for 3 consecutive days increased the SOD activity in comparison with the Cis-administered mice (*P*<0.01) (Figure 3A). Furthermore, administration of Suma at both doses (0.1 and 0.3 mg/kg) significantly increased the activity of GPx in comparison with the Cis-administered mice (*P*<0.01 in all groups) (Figure 3B).

***Renal pathology***

The kidney samples of control group presented normal kidney morphology. In this group, the lumen of the collecting tubules (bent arrow) were obvious and no evidence of tubular injury such as cast, necrosis, degeneration and vacuolation was observed (Figure 4A; Table 1). The kidney sample of Cis group displayed massive tubular injury in the form of tubular degeneration (lightning bolt), tubular cells vacuolation (line arrow), tubular necrosis (white arrow) and cast (triangle) (*P*<0.05 for tubular cells vacuolation, *P*<0.01 for cast and *P*<0.001 for tubular necrosis and tubular degeneration) (Figure 4B; Table 1). Moreover, administration of Suma at the dose of 0.1 mg/kg only reduced the cast (*P*<0.05), but tubular degeneration, cells vacuolation and necrosis were unaffected. In this group, the collecting tubules were more obvious than Cis group (Figure 4C; Table 1). Furthermore, Suma (0.3 mg/kg) effectively reduced the Cis-induced tubular injury and all tubular injury parameters were reduced in these groups (*P*<0.05 for tubular cells vacuolation, tubular necrosis and tubular degeneration and *P*<0.01 for cast). In Suma 0.3 group, the lumen of collecting tubules were obvious, but tubular cells vacuolation was observed. Also, there was no evidence of tubular degeneration, tubular cells vacuolation, tubular necrosis and cast (Figure 4D; Table 1). 

## Discussion

In the current study, the effect of Suma treatment on nephrotoxicity induced by Cis was investigated. Our results demonstrated that IP administration of Cis causes considerable renal injury as evidenced by increase in the levels of BUN and Cr. In line with previous studies, after injection of Cis with the mentioned dose, the MDA level was increased and the SOD and GPx activity were reduced in renal tissue (20, 21). Moreover, the results of renal pathological assessment correlated with changes in biochemical indices. It was also observed that coadministration of Suma (especially 0.3 mg/kg, IP) and Cis prevents nephrotoxicity in mice through decreasing the levels of MDA, Cr and BUN as well as increasing the activities of GPx and SOD. Moreover, IP administration of Suma significantly mitigated the Cis-induced pathological changes of the renal tissues. 

We used BUN and Cr levels as common biochemical tests for evaluation of the kidney functions. A large body of evidence has implicated that Cis-induced elevation of Cr and BUN levels are associated with the increase in the ROS formation (superoxide and hydroxyl radicals) in the kidney tissue (5, 21). On the other hand, Cis increases the production of NO in the kidney (22). It has been found that antioxidant substances such as lycopene and ellagic acid attenuate the elevation of Cr and BUN levels in Cis-induced nephrotoxicity (20, 21, 23). Moreover, it was reported that Suma scavenges superoxide and hydroxyl radicals as well as NO (13). Furthermore, it is well-established that Suma blocks the NO pathway, which is a major mechanism underlying the anti-migraine properties of Suma (24). Accordingly, it seems that Suma may exert its nephroprotective effects via attenuation of the renal oxidative stress.

We observed that Suma signiﬁcantly attenuates MDA in kidney of mice treated with Cis. Overproduction of free radicals plays a critical role in nephrotoxicity induced by Cis, which is associated with increase of oxidative stress parameters in renal tissue such as MDA (1, 25, 26). MDA is a confirmed biomarker for cellular damage (27). It is well-documented that Suma could inhibit pro-oxidative enzymes such as inducible nitric oxide synthase (iNOS) and lipid peroxidase (28). Furthermore, Suma is a strong antioxidant compound, which directly neutralizes free radicals like hydroxyl and superoxide radicals (29). Hence, Suma may reduce MDA directly by reduction of lipid peroxidation and/or indirectly by suppression of free radicals production.

SOD catalyzes the conversion of superoxide radical to H_2_O_2_. Then, H_2_O_2_ is detoxified in cells by GPx (30). Cis causes the oxidative stress, which leads to reduction of renal antioxidant capacity (20, 21). It has been shown that inhibition of the antioxidant defense mechanisms is involved in pathogenesis and progression of migraine; this implies that the therapeutic effects of Suma on migraine might be mediated through increased activity of brain antioxidant defense (31, 32). For the first time, we revealed that Suma administration (more potently 0.3 mg/kg) increases the renal enzymes of antioxidant system such as GPx and SOD.

Our histopathological findings are in parallel with the biochemical results. Cis causes renal morphological and functional lesions via overproduction of free radicals (19, 33, 34). Moreover, previous studies confirm that Cis mainly induces tubular damage (35-38). We also confirmed that Cis causes cast, tubular necrosis, tubular cells vacuolation and tubular degeneration. The nephroprotective effects of Suma were also confirmed by pathological evaluations, which indicated significant attenuation in pathological indices in Cis-administered mice. 

It has been reported that Suma induces adverse effects within the central nervous system (CNS) such as memory impairment, anxiety and panic disorder (9). On the other hand, it has been found that Suma exerts protective effects on peripheral neuropathy induced by vincristine (39). 

## Conclusion

For the first time, we showed that Suma has nephroprotective effect in Cis-induced nephrotoxicity. According to the results of our study, it can be concluded that Suma may protect the cancer patients against the nephrotoxicity of Cis. However, more studies are needed to reveal the precise molecular mechanisms underlying Suma effects on Cis-induced nephrotoxicity.

## References

[1] Yao X, Panichpisal K, Kurtzman N, Nugent K (2007). Cisplatin nephrotoxicity: a review. Am J Med Sci..

[2] Sharp CN, Siskind LJ (2017). Developing better mouse models to study cisplatin-induced kidney injury. Am J Physiol Renal Physiol.

[3] Ozkok A, Edelstein CL (2014). Pathophysiology of cisplatin-induced acute kidney injury. Biomed Res Int.

[4] Kaeidi A, Rasoulian B, Hajializadeh Z, Pourkhodadad S, Rezaei M (2013). Cisplatin toxicity reduced in human cultured renal tubular cells by oxygen pretreatment. Ren fail.

[5] Miller RP, Tadagavadi RK, Ramesh G, Reeves WB (2010). Mechanisms of Cisplatin nephrotoxicity. Toxins (Basel).

[6] Rasoulian B, Kaeidi A, Rezaei M, Hajializadeh Z (2017). Cellular preoxygenation partially attenuates the antitumoral effect of cisplatin despite highly protective effects on renal epithelial cells. Oxid Med Cell Longev.

[7] Boroushaki MT, Rajabian A, Farzadnia M, Hoseini A, Poorlashkari M, Taghavi A (2015). Protective effect of pomegranate seed oil against cisplatin-induced nephrotoxicity in rat. Ren Fail.

[8] Rasoulian B, Kaeidi A, Pourkhodadad S, Dezfoulian O, Rezaei M, Wahhabaghai H (2014). Effects of pretreatment with single-dose or intermittent oxygen on Cisplatin-induced nephrotoxicity in rats. Nephrourol Mon.

[9] Dechant KL, Clissold SP (1992). Sumatriptan. Drugs.

[10] Ikeda Y, Jimbo H, Shimazu M, Satoh K (2002). Sumatriptan scavenges superoxide, hydroxyl, and nitric oxide radicals: in vitro electron spin resonance study. Headache.

[11] Carmichael NM, Charlton MP, Dostrovsky JO (2008). Activation of the 5-HT1B/D receptor reduces hindlimb neurogenic inflammation caused by sensory nerve stimulation and capsaicin. Pain.

[12] Durham PL, Russo AF (2003). Stimulation of the calcitonin gene-related peptide enhancer by mitogen-activated protein kinases and repression by an antimigraine drug in trigeminal ganglia neurons. J Neurosci.

[13] Ikeda Y, Jimbo H, Shimazu M, Satoh K (2002). Sumatriptan scavenges superoxide, hydroxyl, and nitric oxide radicals: in vitro electron spin resonance study. Headache.

[14] Whiting MV, Cambridge D (1995). Canine renovascular responses to sumatriptan and 5-carboxamidotryptamine: modulation through endothelial 5-HT1-like receptors by endogenous nitric oxide. Br J Pharmacol.

[15] Vause CV, Durham PL (2012). Identification of cytokines and signaling proteins differentially regulated by sumatriptan/naproxen. Headache.

[16] Vera-Portocarrero LP, Ossipov MH, King T, Porreca F (2008). Reversal of inflammatory and noninflammatory visceral pain by central or peripheral actions of sumatriptan. Gastroenterology.

[17] Ramesh G, Reeves WB (2005). p38 MAP kinase inhibition ameliorates cisplatin nephrotoxicity in mice. Am J Physiol Renal Physiol.

[18] Dehnamaki F, Karimi A, Pilevarian AA, Fatemi I, Hakimizadeh E, Kaeidi A (2018). Treatment with troxerutin protects against cisplatin-induced kidney injury in mice. Acta Chir Belg.

[19] Ehsani V, Amirteimoury M, Taghipour Z, Shamsizadeh A, Bazmandegan G, Rahnama A (2017). Protective effect of hydroalcoholic extract of Pistacia vera against gentamicin-induced nephrotoxicity in rats. Ren Fail.

[20] Atessahin A, Ceribasi AO, Yuce A, Bulmus O, Cikim G (2007). Role of ellagic acid against cisplatin-induced nephrotoxicity and oxidative stress in rats. Basic Clin Pharmacol Toxicol.

[21] Atessahin A, Yilmaz S, Karahan I, Ceribasi AO, Karaoglu A (2005). Effects of lycopene against cisplatin-induced nephrotoxicity and oxidative stress in rats. Toxicology.

[22] Wang Z, Li YF, Han XY, Sun YS, Zhang LX, Liu W (2018). Kidney protection effect of ginsenoside re and its underlying mechanisms on cisplatin-induced kidney injury. Cell Physiol Biochem.

[23] Chandrasekara N, Shahidi F (2011). Antioxidative potential of cashew phenolics in food and biological model systems as affected by roasting. Food Chem.

[24] Akerman S, Williamson DJ, Kaube H, Goadsby PJ (2002). The effect of anti-migraine compounds on nitric oxide-induced dilation of dural meningeal vessels. Eur J Pharmacol.

[25] Chirino YI, Sanchez-Gonzalez DJ, Martinez-Martinez CM, Cruz C, Pedraza-Chaverri J (2008). Protective effects of apocynin against cisplatin-induced oxidative stress and nephrotoxicity. Toxicology.

[26] Saleh S, El-Demerdash E (2005). Protective effects of L-arginine against cisplatin-induced renal oxidative stress and toxicity: role of nitric oxide. Basic Clin Pharmacol Toxicol.

[27] Priyamvada S, Priyadarshini M, Arivarasu N, Farooq N, Khan S, Khan SA (2008). Studies on the protective effect of dietary fish oil on gentamicin-induced nephrotoxicity and oxidative damage in rat kidney. Prostaglandins Leukot Essent Fatty Acids.

[28] Bulboaca AE, Bolboaca SD, Stanescu IC, Sfrangeu CA, Porfire A, Tefas L (2018). The effect of intravenous administration of liposomal curcumin in addition to sumatriptan treatment in an experimental migraine model in rats. Int J Nanomedicine.

[29] Rajagopalan G, Chandrasekaran SP, Carani Venkatraman A (2017). Troxerutin attenuates diet-induced oxidative stress, impairment of mitochondrial biogenesis and respiratory chain complexes in mice heart. Clin Exp Pharmacol Physiol.

[30] Tabara LC, Poveda J, Martin-Cleary C, Selgas R, Ortiz A, Sanchez-Nino MD (2014). Mitochondria-targeted therapies for acute kidney injury. Expert Rev Mol Med.

[31] Ferroni P, Barbanti P, Della-Morte D, Palmirotta R, Jirillo E, Guadagni F (2017). Redox mechanisms in migraine: novel therapeutics and dietary interventions. Antioxid Redox Signal.

[32] Mehrzadi S, Fatemi I, Malayeri AR, Khodadadi A, Mohammadi F, Mansouri E (2018). Ellagic acid mitigates sodium arsenite-induced renal and hepatic toxicity in male Wistar rats. Pharmacol Rep.

[33] Ghaznavi H, Fatemi I, Kalantari H, Hosseini Tabatabaei SMT, Mehrabani M, Gholamine B (2017). Ameliorative effects of gallic acid on gentamicin-induced nephrotoxicity in rats. J Asian Nat Prod Res.

[34] Goudarzi M, Khodayar MJ, Hosseini Tabatabaei SMT, Ghaznavi H, Fatemi I, Mehrzadi S (2017). Pretreatment with melatonin protects against cyclophosphamide-induced oxidative stress and renal damage in mice. Fundam Clin Pharmacol.

[35] Nho JH, Jung HK, Lee MJ, Jang JH, Sim MO, Jeong DE (2018). Beneficial effects of cynaroside on cisplatin-induced kidney injury in vitro and in vivo. Toxicol Res.

[36] Soni H, Kaminski D, Gangaraju R, Adebiyi A (2018). Cisplatin-induced oxidative stress stimulates renal Fas ligand shedding. Ren Fail.

[37] Zhou J, Fan Y, Zhong J, Huang Z, Huang T, Lin S (2018). TAK1 mediates excessive autophagy via p38 and ERK in cisplatin-induced acute kidney injury. J Cell Mol Med.

[38] Li J, Gui Y, Ren J, Liu X, Feng Y, Zeng Z (2016). Metformin protects against cisplatin-induced tubular cell apoptosis and acute kidney injury via AMPkalpha-regulated autophagy induction. Sci Rep.

[39] Khalilzadeh M, Panahi G, Rashidian A, Hadian MR, Abdollahi A, Afshari K (2018). The protective effects of sumatriptan on vincristine - induced peripheral neuropathy in a rat model. Neurotoxicology.

